# The Impact of Appendectomy in Clostridium difficile Infection and Length of Hospital Stay

**DOI:** 10.7759/cureus.10342

**Published:** 2020-09-09

**Authors:** Rajesh Essrani, Dany Saturno, Shehriyar Mehershahi, Rajesh Kumar Essrani, Muhammad Rajib Hossain, Shri Jai Kirshan Ravi, Andrea Berger, Asif Mehmood

**Affiliations:** 1 Internal Medicine, Geisinger Medical Center, Danville, USA; 2 Internal Medicine, Lehigh Valley Health Network, Allentown, USA; 3 General Internal Medicine, BronxCare Health System, Bronx, USA; 4 Gastroenterology, BronxCare Health System, Bronx, USA; 5 Internal Medicine, Bronx Lebanon Hospital Center, Bronx, USA; 6 Hospital Medicine, Geisinger Medical Center, Danville, USA; 7 General Internal Medicine, Guthrie Robert Packer Hospital, Sayre, USA; 8 Biostatistics, Geisinger Medical Center, Danville, USA; 9 Internal Medicine, Abington Hospital - Jefferson Health, Abington, USA

**Keywords:** clostridium difficile, clostridium difficile infection, abdominal pain, antibiotics

## Abstract

Introduction

We aim to investigate Clostridium difficile infection (CDI) recurrence, severity, complications, and length of hospital stay in patients with and without prior history of appendectomy who were admitted to the hospital with CDI.

Method

We analyzed retrospective data for 862 patients, 18 years and older, with C. difficile inpatients diagnosed between January 1, 2017 and December 31, 2018 and sorted into two groups, with or without prior appendicectomy, to look for outcomes such as recurrence, hospital stay, complications, and related death in each group and use statistical analysis for comparison.

Result

There were 862 patients admitted with CDI, of which 122 (14.2%) had a prior history of appendectomy and 740 (85.8%) did not. Patients with an appendectomy prior were older (median age of 75 vs. 69, p = 0.0033) and had a higher proportion of females (68.9% vs. 53.6%, p = 0.0017).

C. difficile recurrence in prior appendicectomy group vs. no appendectomy group was 12.3% and 9.3%, respectively, but no statistical difference was noted (p = 0.28). Also, there was no statistical difference in complications like ileus, colectomy, and mortality related to CDI in both groups. However, patients with appendectomies had significantly shorter hospital stays during C. difficile admission compared to patients without appendectomies (median of six days vs. seven days, p = 0.0014).

Conclusion

Our study shows that there is no statistical difference in the recurrence, severity, and complications of CDI in the presence or absence of the appendix but remarkably noted that people with prior appendicectomy had a shorter hospital stay.

## Introduction

Clostridium difficile infection (CDI) is one of the most common infectious etiology of hospital-acquired (nosocomial) infections in hospitalized patients [1]. The generally accepted theory for the pathogenesis of C. difficile involves the disruption of normal intestinal flora, typically in the setting of prior antibiotic use, which allows C. difficile to proliferate [2]. The clinical spectrum of CDI ranges from mild diarrhea to fulminant colitis with shock, ileus, toxic megacolon, and death [3,4]. Recurrent CDI is defined by the resolution of CDI symptoms while on appropriate therapy, followed by the reappearance of symptoms within two to eight weeks after treatment has been stopped [1]. There are several risk factors for CDI and recurrence, such as age greater than 65 years, prolonged hospital stays, use of antibiotics other than C. difficile therapy, use of antacid, or immunosuppressive medications [5-7]. These risk factors suggest that the interaction between the host immunity and colonic flora disruption plays a significant role in CDI severity, recurrence, and length of stay.

The vermiform appendix is theorized to serve as a microbial reservoir and plays a vital role in the maintenance of colonic bacteria after serious gut infections [8,9]. The idea that the appendix has the ideal environment to maintain enteric bacteria in biofilms is reasonable, and its role in immune protection may be based upon providing normal colonic flora in times of need [8,9].

We aim to investigate CDI recurrence, severity, complications, and length of hospital stay in patients with and without prior history of appendectomy who were admitted to the hospital with CDIs. 

This article was presented as a poster presented at Digestive Disease Week, Chicago, IL, USA (Essrani, R. Mehershahi, S. Hossain, MR. Ravi, SJK, Berger, A. Mehmood, A. The Impact of Appendectomy in Clostridium Difficile Infection and Length of Hospital Stay; May 2020).

## Materials and methods

We analyzed retrospective data for 862 patients, 18 years and older, with C. difficile inpatients diagnosed between January 1, 2017 and December 31, 2018. The study was approved by the Institutional Review Board. The requirement of informed consent was waived at the time of approval due to the retrospective design. The charts of 87 patients who had been identified with a reoccurrence of C. difficile after their index admission were manually reviewed by the investigator to confirm the reoccurrence and to assess if a patient was treated with a fecal microbiota transplant (FMT). Baseline characteristics were compared using Student’s t-tests, Wilcoxon rank-sum tests, or Pearson’s chi-square and Fisher’s exact tests. Mortality was compared using Kaplan-Meier curves and log-rank tests. Due to high post-discharge patient mortality complications of C. difficile and reoccurrence were analyzed using Fine-Gray subdistribution hazard models where death was treated as a competing risk. Linear regression models with a natural log transformation of hospital length of stay were used to estimate the percentage decrease in stay days for patients with a prior appendectomy compared to those without. Results are also reported from a multivariable model adjusted for age and sex.

## Results

There were 862 patients admitted with CDIs, of which 122 (14.2%) had a prior history of appendectomy and 740 (85.8%) did not. The median age at the time of admission was 70 years and 55.8% were female. Patients with an appendectomy prior were older (median age of 75 years vs. 69, p = 0.0033) and had a higher proportion of females (68.9% vs. 53.6%, p = 0.0017). Groups were similar with respect to body mass index (BMI), lab values, blood pressure, and comorbid conditions (Table 1).

**Table 1:** Comparison of Baseline Demographic and Clinical Characteristics of Patients With and Without Prior History of Appendectomy C. difficile, Clostridium difficile; IQR, interquartile range; BMI, body mass index; WBC, white blood cell; SD, standard deviation.

	All Patients	Appendectomy Prior	No Appendectomy	P-Value
Number of patients, n	862	122	740	
Age at C. difficile diagnosis, median (IQR)	70 (59, 80)	75 (63, 84)	69 (58, 79)	0.0033
BMI, median (IQR), n missing = 13	26.8 (22.7, 32.4)	26.9 (23.2, 32.8)	26.8 (22.6, 32.4)	0.5620
WBC, median (IQR), n missing = 2	11.5 (7.7, 16.4)	10.7 (7.0, 15.4)	11.6 (7.8, 16.5)	0.1073
Creatinine, median (IQR), n missing = 2	1.1 (0.8, 1.8)	1.2 (0.9, 1.9)	1.1 (0.8, 1.8)	0.2593
Systolic blood pressure, mean (SD)	125.0 (26.5)	126.8 (26.3)	124.7 (26.5)	0.4323
Diastolic blood pressure, mean (SD)	70.3 (17.7)	70.2 (15.3)	70.3 (18.1)	0.9418
Sex, n (%)				0.0017
Female	481 (55.8%)	84 (68.9%)	397 (53.6%)	
Male	381 (44.2%)	38 (31.1%)	343 (46.4%)	
Years from appendectomy to C. difficile, median (IQR)	N/A	8.6 (3.8, 14.3)	N/A	N/A
Year of C. difficile diagnosis, n (%)				0.5811
2017	209 (24.2%)	32 (26.2%)	177 (23.9%)	
2018	653 (75.8%)	90 (73.8%)	563 (76.1%)	
History of diabetes, n (%)	337 (39.1%)	54 (44.3%)	283 (38.2%)	0.2068
History of malignancy, n (%)	302 (35.0%)	48 (39.3%)	254 (34.3%)	0.2816
History of cirrhosis, n (%)	41 (4.8%)	4 (3.3%)	37 (5.0%)	0.4079

There was no statistical difference in complications like ileus, colectomy, and recurrence of CDI in both groups. A total of 33 patients (27.3%) with a history of appendectomy died during the study period compared to 264 patients (35.9%) without an appendectomy (log-rank p-value = 0.0736). Patients with appendectomies had significantly shorter hospital stays during C. difficile admission compared to those without appendectomies (median of six days vs. seven days, p = 0.0014) (Table 2). 

**Table 2:** Comparison of Outcomes for Patients With and Without Prior History of Appendectomy IQR, interquartile range.

	All Patients	Appendectomy Prior	No Appendectomy	P-Value
Number of patients, n	862	122	740	
Ileus, n (%)	84 (9.7%)	14 (11.5%)	70 (9.5%)	0.5038
Megacolon, n (%)	0 (0.0%)	0 (0.0%)	0 (0.0%)	N/A
Colectomy, n (%)	16 (1.9%)	2 (1.6%)	14 (1.9%)	0.8388
Complication (iIleus or colectomy), n (%)	92 (10.7%)	14 (11.5%)	78 (10.5%)	0.7831
Clostridium difficile reoccurrence, n (%)	84 (9.8%)	15 (12.3%)	69 (9.3%)	0.2805
Deceased, n (%), n missing = 5	297 (34.7%)	33 (27.3%)	264 (35.9%)	0.0736
Length of stay days, median (IQR)	6 (4, 11)	6 (4, 8)	7 (4, 11)	0.0014

Patients with a prior appendectomy stayed 23.4% fewer days in the hospital compared to patients without appendectomies (95% CI: -33.4%, -9.6%). After adjusting for age and sex, patients who had a prior appendectomy stayed 23.3% fewer days in the hospital (95% CI: -34.3%, -10.6%).

A total of 84 patients experienced a reoccurrence of C. difficile, of which 15 (17.9%) had appendectomies prior and 69 (82.1%) did not. One appendectomy patient and three non-appendectomy patients received an FMT. All four patients received an OpenBiome transplant. Patients did not differ with respect to baseline characteristics or if they received FMT (Table 3).

**Table 3:** Comparison of Patients With and Without Prior History of Appendectomy Who Experienced Reoccurrence C. difficile, Clostridium difficile; BMI, body mass index; IQR, interquartile range; WBC, white blood cell; SD, standard deviation; FMT, fecal microbiota transplant.

	All Patients With Reoccurrence	Appendectomy Prior	No Appendectomy	P-Value
Number of patients, n	84	15	69	
Age at C. difficile diagnosis, median (IQR)	74 (61, 83)	76 (61, 85)	72 (61, 82)	0.4200
BMI, median (IQR), n missing = 2	26.7 (22.7, 32.5)	27.3 (26.0, 34.7)	26.7 (22.4, 32.0)	0.2569
WBC, median (IQR), n missing = 1	11.7 (7.7, 16.6)	10.7 (6.9, 14.6)	11.8 (7.8, 17.1)	0.7107
Creatinine, median (IQR), n missing = 1	1.2 (0.8, 2.1)	1.6 (0.9, 2.2)	1.2 (0.8, 1.9)	0.2447
Systolic blood pressure, mean (SD)	125.7 (23.4)	130.3 (23.1)	124.7 (23.5)	0.4040
Diastolic blood pressure, mean (SD)	68.1 (17.4)	65.6 (19.1)	68.7 (17.1)	0.5391
Sex, n (%)				0.4701
Female	49 (58.3%)	10 (66.7%)	39 (56.5%)	
Male	35 (41.7%)	5 (33.3%)	30 (43.5%)	
Years from appendectomy to C. difficile, median (IQR)	N/A	5.9 (2.0, 15.4)	N/A	N/A
Year of C. difficile diagnosis, n (%)				0.7500
2017	23 (27.4%)	3 (20.0%)	20 (29.0%)	
2018	61 (72.6%)	12 (80.0%)	49 (71.0%)	
History of diabetes, n (%)	45 (53.6%)	10 (66.7%)	35 (50.7%)	0.2618
History of malignancy, n (%)	37 (44.0%)	6 (40.0%)	31 (44.9%)	0.7275
History of cirrhosis, n (%)	3 (3.6%)	1 (6.7%)	2 (2.9%)	0.4501
History of FMT, n (%)	4 (4.8%)	1 (6.7%)	3 (4.3%)	0.5520
OpenBiome FMT, n (%)	4 (100.0%)	1 (100.0%)	3 (100.0%)	N/A
Mode of FMT delivery, n (%)				1.0000
Oral capsule	0 (0.0%)	0 (0.0%)	0 (0.0%)	
Colonoscopy	3 (75.0%)	1 (100.0%)	2 (66.7%)	
Upper endoscopy	1 (25.0%)	0 (0.0%)	1 (33.3%)	
C. difficile reoccurrence after FMT, n (%)	1 (25.0%)	0 (0.0%)	1 (33.3%)	1.0000

## Discussion

C. difficile colitis is a highly prevalent infection cause by an anaerobic, gram-positive, spore-forming bacteria known as C. difficile. The CDI has a wide spectrum of clinical presentation ranging from non-severe, severe to a fulminant colitis [3,4,10]. An epidemiologic study conducted in 2011 identified 453,000 cases and 29,000 deaths associated with CDI [11].

It has been hypothesized that the appendix serves as a microbial reservoir and plays a vital role in the maintenance of colonic bacteria after serious gut infections; therefore, there is the impression that patients who underwent appendectomy are at a higher risk of developing recurrent infection but not initial onset of C. difficile-associated colitis [12].

Current data demonstrate conflicting evidence on repercussions of a prior appendectomy on CDI. According to Im et al., patients who had an appendectomy done were found to have an increased risk of recurrent CDI and were also associated with a more severe clinical course [13]. In contrast, Franko et al. found no effect of appendectomy status on recurrence of CDI [14]. The aim of our study was to determinate CDI recurrence, severity, complications, and length of hospital stay in patients with and without prior history of appendectomy who were admitted to the hospital with CDIs.

As mentioned in the results, among 862 patients who were included in the study, only 122 patients had an appendectomy done in the past, and no statistical difference in the incidence of recurrence was found between the two groups (15 patients with prior appendectomy who represent 12.3% of this subgroup and 69 patients without appendectomy who represent 9.3% of this subgroup had recurrent CDI). Older age, renal failure, and underlying comorbidities are frequent risk factors for complicated CDI [15]. In our subgroup with prior appendectomy, the median age was 75 years, which was higher compared to the non-appendectomy group that was 69 years. This could be a confounding factor that may have created a difference between the two groups, contraintuitive to what would be expected the group post appendectomy would be at a higher risk for recurrence (explained by their age and by appendectomy itself) compared to non-appendectomy group, but our results as mentioned before showed no difference. Data from multiple studies have suggested a possible protective role of the appendix.

The appendix has been described as a well-known site of production of IgA, the predominant immunoglobulin in the gut associated lymphoid tissue (GALT) system [16]. Several studies have pointed to an association between IgA levels and C. difficile colonization and infection [17]. According to Azrad et al., serum IgA was shown to block inflammatory response by suppression of phagocytosis, chemotaxis, and cytokine production [18]. Considering this information, it would be expected that the non-appendectomy patients would have a less severe form of this infection and therefore have a shorter length of stay compared to the appendectomy patients. In contrast, our study found that patients with appendectomies had significantly shorter hospital stays compared to patients without appendectomies. We can imply this difference could be related to faster response to treatment in the case of appendectomized patients. Because of the lack of good evidence on the subject, we suggest further study on the matter.

The study has several limitations as it was a retrospective study, with a relatively small sample size that reflects only those that came in our hospital from January 1, 2017 and December 31, 2018. Due this we were not able to do stratified analysis to rule out other confounding factors, such as difference on age, proton pump inhibitor (PPI) consumption, enteral feeding, and gastrointestinal surgery.

## Conclusions

Despite what has been described in previous studies, we found there is no statistical difference in the recurrence, severity, and complications of CDI in the presence or absence of the appendix but remarkably noted that people with prior appendicectomy had a shorter hospital stay. Most of literature on this subject is based on observational data and retrospective studies that limits external validity of the data; further, prospective studies are warranted to validate the result.

## References

[1] McDonald LC, Gerding DN, Johnson S (2018). Clinical practice guidelines for Clostridium difficile infection in adults and children: 2017 Update by the Infectious Diseases Society of America (IDSA) and Society for Healthcare Epidemiology of America (SHEA). Clin Infect Dis.

[2] Barbut F, Petit JC (2001). Epidemiology of Clostridium difficile-associated infections. Clin Microbiol Infect.

[3] Monaghan T, Boswell T, Mahida YR (2009). Recent advances in Clostridium difficile-associated disease. Postgrad Med J.

[4] Fekety R, McFarland LV, Surawicz CM, Greenberg RN, Elmer GW, Mulligan ME (1997). Recurrent Clostridium difficile diarrhea: characteristics of and risk factors for patients enrolled in a prospective, randomized, double-blinded trial. Clin Infect Dis.

[5] Aslam S, Musher DM (2006). An update on diagnosis, treatment, and prevention of Clostridium difficile-associated disease. Gastroenterol Clin North Am.

[6] Hu MY, Katchar K, Kyne L (2009). Prospective derivation and validation of a clinical prediction rule for recurrent Clostridium difficile infection. Gastroenterology.

[7] T. Monaghan, T. Boswell, Y.R Y.R Recent advances in Clostridium difficile-associated disease. Postgrad Med J.

[8] Randal Bollinger R, Barbas AS, Bush EL, Lin SS, Parker W (2007). Biofilms in the large bowel suggest an apparent function of the human vermiform appendix. J Theor Biol.

[9] Ansaloni L, Catena F, Pinna AD (2009). What is the function of the human vermiform appendix?. Evolution-based surgery: a new perspective in the Darwinian year 2009. Eur Surg Res.

[10] Surawicz CM, Brandt LJ, Binion DG (2013). Guidelines for diagnosis, treatment, and prevention of Clostridium difficile infections. Am J Gastroenterol.

[11] Lessa FC, Mu Y, Bamberg WM (2015). Burden of Clostridium difficile infection in the United States. N Engl J Med.

[12] Sanders NL, Bollinger RR, Lee R, Thomas S, Parker W (2013). Appendectomy and Clostridium difficile colitis: relationships revealed by clinical observations and immunology. World J Gastroenterol.

[13] Im GY, Modayil RJ, Lin CT, Geier SJ, Katz DS, Feuerman M, Grendell JH (2011). The appendix may protect against Clostridium difficile recurrence. Clin Gastroenterol Hepatol.

[14] Franko J, Ferrel B, Pierson P (2020). Influence of prior appendectomy and cholecystectomy on Clostridioides difficile infection recurrence and mortality. Am J Surg.

[15] Abou Chakra CN, Pepin J, Sirard S, Valiquette L (2014). Risk factors for recurrence, complications and mortality in Clostridium difficile infection: a systematic review. PLoS One.

[16] Dasso JF, Obiakor H, Bach H, Anderson AO, Mage RG (2000). A morphological and immunohistological study of the human and rabbit appendix for comparison with the avian bursa. Dev Comp Immunol.

[17] Bridgman SL, Konya T, Azad MB (2016). High fecal IgA is associated with reduced Clostridium difficile colonization in infants. Microbes Infect.

[18] Azrad M, Hamo Z, Tkhawkho L, Peretz A (2018). Elevated serum immunoglobulin A levels in patients with Clostridium difficile infection are associated with mortality. Pathog Dis.

